# Role of HDAC3-miRNA-CAGE Network in Anti-Cancer Drug-Resistance

**DOI:** 10.3390/ijms20010051

**Published:** 2018-12-23

**Authors:** Yoojung Kwon, Youngmi Kim, Hyun Suk Jung, Dooil Jeoung

**Affiliations:** 1 Department of Biochemistry, College of Natural Sciences, Kangwon National University, Chunchon 24341, Korea; kkwon89@kangwon.ac.kr (Y.K.); hsjung@kangwon.ac.kr (H.S.J.); 2 Institute of New Frontier Research, College of Medicine, Hallym University, Chunchon 24251, Korea; kym8389@hanmail.net

**Keywords:** anti-cancer drug-resistance, histone deacetylase-3, cancer/testis antigen CAGE, epidermal growth factor receptor, micro RNAs, molecular network

## Abstract

Histone modification is associated with resistance to anti-cancer drugs. Epigenetic modifications of histones can regulate resistance to anti-cancer drugs. It has been reported that histone deacetylase 3 (HDAC3) regulates responses to anti-cancer drugs, angiogenic potential, and tumorigenic potential of cancer cells in association with cancer-associated genes (CAGE), and in particular, a cancer/testis antigen gene. In this paper, we report the roles of microRNAs that regulate the expression of HDAC3 and CAGE involved in resistance to anti-cancer drugs and associated mechanisms. In this review, roles of HDAC3-miRNAs-CAGE molecular networks in resistance to anti-cancer drugs, and the relevance of HDAC3 as a target for developing anti-cancer drugs are discussed.

## 1. HDAC3 as a Target for Development of Anti-Cancer Drugs

Unlike many other histone deacetylases (HDACs), HDAC3 shows ubiquitous expression [1]. Nuclear Receptor Corepressor (N-CoR) and silencing mediator of retinoid and thyroid hormone receptors (SMRT) bind to HDAC3 and exert transcriptional repression [2]. The N-CoR-HDAC3 complex inhibits the c-jun N-terminal kinase (JNK) pathway through the GPS2 subunit [3]. HDAC3 deacetylates relA, subunit of nuclear factor-kB (NF-κB), to repress NF-κB activity [4]. HDAC3 inhibits mitogen-activated protein kinase (MAPK)-dependent activation of activating transcription factor 2 (ATF-2) to repress expression of tumor necrosis factor-α (TNF-α) [5]. These findings suggest that HDAC3 plays an important role in various cellular reactions.

HDAC3 can bind to the promoter sequences of Runt-related transcription factor 2 (Runx 2) and suppress the metastatic potential of colorectal cancers [6]. HDAC3 can also bind to cAMP response element-binding protein (CREB) and decrease the migration potential of metastatic breast cancer cells [7]. HDAC3, a downstream target of AKT serine/threonine kinase (Akt) and glycogen synthase kinase 3 beta (GSK3β), can promote neuronal death [8] and beta cell apoptosis induced by inflammatory cytokines [9]. HDAC3 can also decrease expression of Hypoxia-inducible factor-1 α (HIF-1α) by negative regulation of NF-κB in metastatic breast cancer cells [10] and regulates the signal transducer and activator of transported 3 (Stat3) which inhibits Beclin 1, a prognostic marker and autophagy in non-small cell lung cancer cells [11]. Down-regulation of HDAC3 enhances the tumorigenic potential of lung cancer cells [12]. HDAC3 can also decrease the angiogenic potential of melanoma cells [13]. The role of HDAC3 in cancer development is known to be tissue-specific. It has been reported that liver-specific deletion of HDAC3 can lead to hepatoma by causing genomic instability and DNA damage [14].

Inactivation of HDAC3 by Cre-recombinase can delay cell cycle progression and induce apoptosis in mouse embryonic fibroblast cells (MEFs) [15]. Overexpression of HDAC3 in colorectal cancer cells is known to decrease expression of p21 [16]. Down-regulation or pharmacological inhibition of HDAC3 by novel class I inhibitors, such as 4SC202, BG45, or SBHA, can inhibit cholangiocarcinoma (CCA) growth and promote apoptosis [17]. 

Delphinidin, a specific inhibitor of HDAC3, induces acetylation of p53 and promotes caspase-dependent apoptosis [18]. JNK phosphorylation of HDAC3 leads to increased HDAC3 activity [19]. I-7a, a novel HDAC3 inhibitor, acetylates p53 and p21 expression, which in turn leads to G1 arrest in triple-negative breast cancer (TNBC) [20]. Overexpression of HDAC3 is correlated with poor prognosis in various cancers [21,22]. HDAC3 can decrease expression of p53-upregulated modulator of apoptosis (PUMA), while down-regulation of HDAC3 leads to increased expression of PUMA by p53 [23]. The N-hydroxycinnamamide-based HDAC inhibitor with HDAC1/3 dual selectivity has shown promise as an anti-cancer drug [24]. Thus, HDAC3 can be considered as a target for the development of anti-cancer therapeutics.

## 2. Anti-Cancer Therapy Targeting CSCS in Relation to HDACs 

Cancer stem cells (CSCs) are undifferentiated tumor cells with the capacity for self-renewal [25,26]. HDAC inhibitors can act on CSCs [27,28,29]. Pan-HDAC inhibitors can suppress CSCs via different mechanisms. AR-42 (OSU-HDAC42) is known to promote apoptosis of leukemic stem cells by inhibiting NF-κB and heat shock protein 90 (Hsp90) [30]. Suberoylanilide Hydroxamic Acid (SAHA) and 5-aza-2-deoxycytidine, an inhibitor of DNA methyl transferase I (DNMT1), can inhibit self-renewal and cellular proliferation of pancreatic cancer stem cells [31]. SAHA can reverse cisplatin resistance and decrease CSCs by down-regulating nanog, a marker of cancer stem cells [32].

Hypoxia-inducible factor-1 α (HIF-1α) links inflammation to cancer. Overexpression of HIF-1α indicates poor prognosis [33]. HIF-1α maintains CSCs by inhibiting the negative regulator of Notch1 signaling [34]. HDAC3 binds to HIF-1α and acts as a positive regulator of HIF-1α stability [35]. Arrest-defective-1(ARD1) binds to HIF-1α and mediates acetylation, leading to ubiquitination of HIF-1α [36]. However, HDAC1 [37] and the class II isoforms, HDAC4 and HDAC6 [38] prevents ARD1 from mediating ubiquitination of HIF-1α. Thus, HDAC3 can maintain CSCs by maintaining expression of HIF-1α.

Dysregulation of Stat3 signaling is known to enhance tumorigenic potential of cancer cells by increasing expression levels of angiogenesis markers, such as HIF-1α and the vascular endothelial growth factor (VEGF) [39]. HDAC3 is necessary for STAT3-dependent liver cancer [40]. The NF-κB signaling pathway contributes to the maintenance of glioblastoma stem cells [41]. Glioblastoma stem cells drive anti-cancer drug resistance [41]. NF-κB signaling is accompanied by resistance to the anti-cancer drug Temozolomide (TMZ) in glioblastoma stem cells [42]. RGFP109, a selective inhibitor of HDAC3, can enhance sensitivity to TMZ by inhibiting NF-κB signaling in a TMZ-resistant (TR) GBM cell line [42]. HDAC3 binds to the p65 subunit of NF-κB to inhibit NF-κB [43].

Abexinostat, another pan-HDAC inhibitor, exhibits anti-CSC activities by inducing differentiation [44]. Pan-HDAC inhibitors MC1742 and MC2625 can increase the level of acetylated histone H3 and induce apoptosis in sarcoma cancer stem cells [45]. Down-regulation or inhibition of HDAC3 reduces cholangiocarcinoma (CCA) cell growth and apoptosis [46]. HDACs can regulate transition of non-CSCs into anti-cancer drug-resistant CSCs by chemotherapy [47]. Thus, HDAC inhibitors can restore anti-cancer drug sensitivity by inhibiting CSC plasticity.

## 3. Role of HDAC3 in Anti-Cancer Drug Resistance

Trichostatin A, an inhibitor of HDACs, enhances sensitivity to anti-cancer drugs by decreasing expression of multidrug resistance protein 1(MDR1) [48]. SAHA, an inhibitor of HDAC(s), induces MDR to confer anti-apoptotic effects [49]. These results suggest the important role of HDAC3 in anti-cancer drug resistance.

Taxol-resistant ovarian tumors display altered expression of tubulins [50]. Paclitaxel resistance is accompanied by high expression levels of tubulin β3 in cancer cells such as non-small cell lung cancer cells [51,52]. It has been reported that taxol resistance is due to MAPK activation in colon cancer cells [53]. Taxol resistance is induced by the Phosphoinositide 3-kinase (PI3K)-Akt-JNK signaling pathway in osteosarcoma cells [54]. HDAC3 can inhibit JNK signaling [3] and MAPK activation [5].

**Figure 1:**
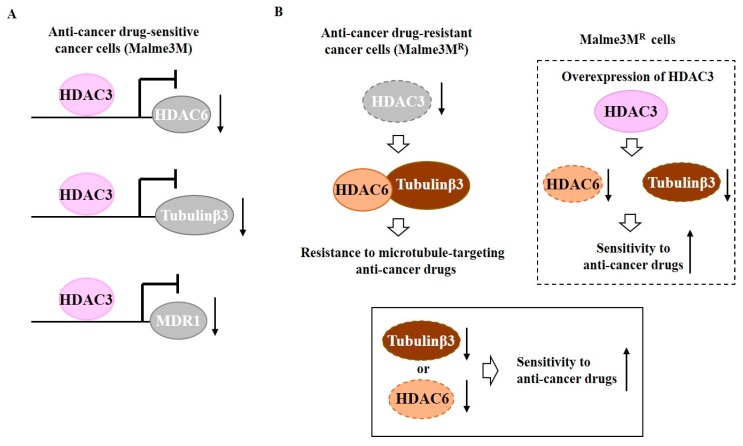
Tubulin β3 serves as a downstream target of histone deacetylase 3 (HDAC3) and mediates resistance to microtubule-targeting drugs. (**A**) In anti-cancer drug-sensitive cancer cells, HDAC3 binds to the promoter sequences of histone deacetylase 6 (HDAC6), tubulin β3, and MDR1. HDAC3 decreases expression levels of HDAC6, tubulin β3, and MDR1. (**B**) In anti-cancer drug-resistant melanoma cells (Malme3M^R^ cells), HDAC6 interacts with tubulin β3. Down-regulation of HDAC3 induces interaction of HDAC6 with tubulin β3. Down-regulation of tubulin β3 or HDAC6 enhances sensitivity of Malme3M^R^ cells to anti-cancer drugs. The T-bar arrows denote inhibition of transcription. The↑rrows denotes increased expression level and ↓arrows denotes decreased expression level.

HDAC3 can bind to promoter sequences of tubulin β3, HDAC6, and MDR1, and negatively regulate expression levels of these proteins [55] (Figure 1A). Down-regulation of HDAC6 or tubulin β3 enhances sensitivity of Malme3M^R^ cells to anti-cancer drugs [55]. HDAC6 shows an interaction with tubulin β3 that is disrupted by HDAC3 [55] (Figure 1B). HDAC6 can interact with beta tubulin in yeast two-hybrid assays and deacetylate tubulin [56]. HDAC3 functions upstream of HDAC6 and tubulin β3 (Figure 1B). Therefore, tubulin β3 is a downstream target of HDAC3 that confers resistance to anti-cancer drugs.

We investigated the role of ubiquitination in the expression regulation of HDAC3. The loss of Seven in Absentia Homolog 2 (SIAH2) is known to increase chemo-sensitivity [57]. E3 ubiquitin ligase activity of SIAH2 is necessary for resistance to death receptor-mediated apoptosis [58]. Inhibition of SIAH2 ubiquitin ligase blocks melanoma tumorigenesis [59]. Loss of SIAH2 suppresses tumorigenesis in a large tumor suppressor kinase 2 (LATS2)-dependent manner in a xenograft mouse model [60]. SIAH2 expression shows an inverse correlation with the tumor grade, p53, and human epidermal growth factor receptor 2 (HER2) [60]. The inhibition of SIAH2 activity reduces metastatic potential, but not tumorigenic potential, via HIF-1α [61]. SIAH2 targets p53 and regulates the acetylation status of p53 [62]. Ski protein stabilizes HDAC3 by binding to SIAH2 [63]. 

**Figure 2:**
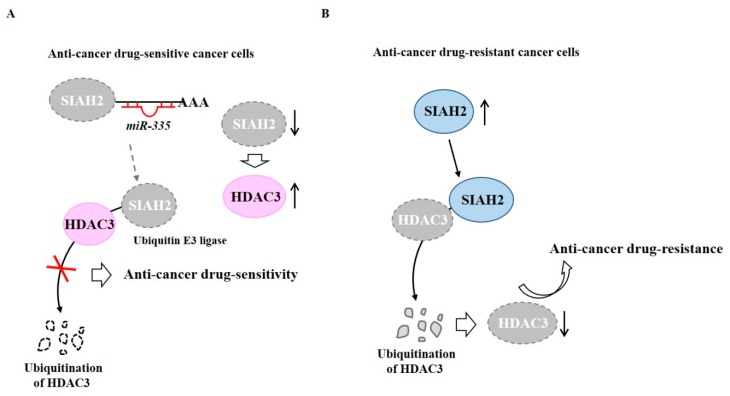
Ubiquitination of HDAC3 leads to decreased expression of HDAC3. (**A**) In anti-cancer drug-sensitive cancer cells, *miR-335* binds to 3′-UTR of *Seven in Absentia Homolog 2 (SIAH2)* (E3 ubiquitin ligase) to decrease expression of SIAH2. However, SIAH2 does not bind to HDAC3. HDAC3 confers sensitivity to anti-cancer drugs. (**B**) In anti-cancer drug-resistant cancer cells, SIAH2 binds to HDAC3 and causes ubiquitination of HDAC3. The lack of HDAC3 confers resistance to anti-cancer drugs. The↑arrows denotes increased expression level and ↓arrows denotes decreased expression level.

TargetScan analysis has predicted that *miR-335* is a negative regulator of SIAH2. *MiR-335* binds to 3′-UTR of *SIAH2* and decreases expression of SIAH2, which in turn increases the expression of HDAC3 to confer anti-cancer drug sensitivity in anti-cancer drug-sensitive melanoma cells, such as Malme3M cells [64] (Figure 2A). HDAC3 may increase expression of *miR-335*. In Malme3M^R^ cells (anti-cancer drug-resistant melanoma cells), SIAH2 can binds to HDAC3, resulting in degradation of HDAC3 by ubiquitination [64] (Figure 2B). Thus, HDAC3 may confer sensitivity to anti-cancer drugs by regulating expression of SIAH2.

## 4. HDAC3-miRNA Network in Angiogenesis and Anti-Cancer Drug Resistance

**Figure 3:**
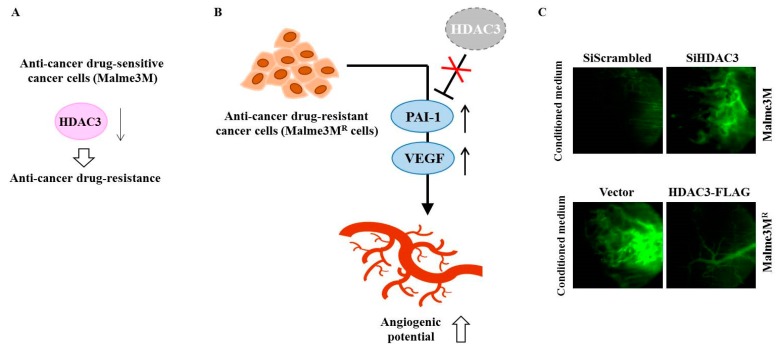
HDAC3 regulates the angiogenic potential of cancer cells. (**A**) In anti-cancer drug-sensitive cancer cells (Malme3M cells), the down-regulation of HDAC3 confers resistance to anti-cancer drugs. (**B**) In anti-cancer drug-resistant cancer cells (Malme3M^R^ cells), decreased expression of HDAC3 leads to increased expression levels of angiogenic factors, such as PAI-1 and VEGF. (**C**) Malme3M cells or Malme3M^R^ cells were transfected with indicated siRNA or construct. Conditioned medium obtained after transfection was mixed with matrigel and subjected to intravital microscopy. The↑arrows denotes increased expression level and ↓arrows denotes decreased expression level.

Anti-cancer drug-resistant phenotypes are under epigenetic regulation [65,66]. Among various HDACs, HDAC3 can inhibit the invasion, migration, and angiogenic potential of hepatic cancer cell lines and melanoma cell lines [67,68]. HDAC3 also confers sensitivity to anti-cancer drugs [67,69] (Figure 3A). Many reports have suggested a close relationship between angiogenic potential and anti-cancer drug resistance [68]. HDAC3 inhibits the angiogenic potential of cancer cells by decreasing expression levels of angiogenic factors, such as VEGF and plasminogen activator inhibitor-1 (PAI-1) [22] (Figure 3B). VEGF and HDAC3 can form a negative feedback loop and regulate endothelial cell tube formation [13]. Down-regulation of HDAC3 enhances angiogenic potential of Malme3M cells, while overexpression of HDAC3 decreases the angiogenic potential of Malme3M^R^ cells [13] (Figure 3C).

**Figure 4:**
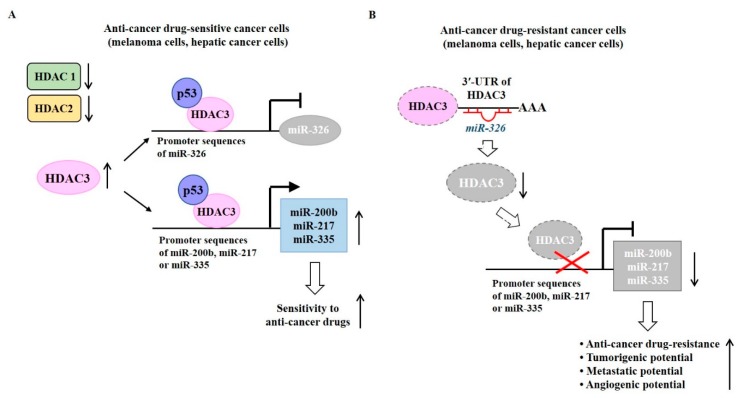
HDAC3-miRNA network in anti-cancer drug resistance. (**A**) In anti-cancer drug-sensitive cancer cells, the HDAC3-p53 complex binds to promoter sequences of miR-326 and decreases expression of miR-326. The HDAC3-p53 complex binds to promoter sequences of miR-200b, miR-217, and miR-335 and increases expression levels of these miRNAs. (**B**) In anti-cancer drug-resistant cancer cells, miR-326 binds to 3‘-UTR of the *HDAC3* gene to decrease expression of HDAC3. As a result, HDAC3 does not bind to promoter sequences of miR-200b, miR-217, or miR-335. Decreased expression levels of miR-200b, miR-217, and miR-335 can confer anti-cancer drug resistance, enhance tumorigenic potential and metastatic potential, and increase angiogenic potential. The T-bar arrows denote inhibition of transcription and →arrows denote activation of transcription. The ↑arrows denotes increased expression level/increased characteristics and ↓arrows denotes decreased expression level.

Expression levels of HDAC1 and HDAC2 are higher in melanoma cells resistant to anti-cancer drugs than those in melanoma cells sensitive to anti-cancer drugs [64] (Figure 4A). It is probable that HDAC1 and HDAC-2 might be able to confer resistance to anti-cancer drugs. Malme3M^R^ cells show lower expression of HDAC3 than parental sensitive Malme3M cells [64] (Figure 4B). Melanoma cells that are naturally resistant to anti-cancer drugs also showed lower expression of HDAC3 than anti-cancer drug-sensitive melanoma cells [64]. Overexpression of HDAC3 overcomes resistance of Malme3M^R^ cells to anti-cancer drugs [64].

MicroRNAs (miRNAs) are small, non-coding RNAs (21–23 nucleotides) that functions in post-transcriptional regulation of gene expression. MiRNAs can regulate the expression of various oncogenes and tumor suppressor genes [70]. MicroRNA array analyses show differential expression of miRNAs, such as *miR-326*, *miR-200b*, *miR-217*, and *miR-335* between Malme3M cells and Malme3M^R^ cells [64]. *MiR-326* negatively regulated by Notch can decrease the tumorigenic potential of glioma cells [71]. *MiR-326* enhances sensitivity of etoposide (VP-16)-resistant breast cancer cell lines, MCF-7/VP to VP-16 and doxorubicin [72]. *MiR-335* increases the expression of HDAC3 by preventing SIAH2 from inducing ubiquitination of HDAC3 [64]. *MiR-326* directly regulated by HDAC3 (Figure 4A) can regulate responses to anti-cancer drugs [64]. Chromatin immunoprecipitation (ChIP) assays have revealed that HDAC3 can bind to promoter sequences of *miR-200b*, *miR-217*, and *miR-335* in Malme3M cells (Figure 4A). Increased expressions of *miR-200b*, *miR-217*, and *miR-335* confer sensitivity to anti-cancer drugs. In Malme3M^R^cells, *miR-326* binds to 3′-UTR of the *HDAC3* gene and decreases expression of HDAC3 (Figure 4B). Decreased expression of HDAC3 decreases expression levels of *miR-200b*, *miR-217*, and *miR-335* to confer anti-cancer drug resistance (Figure 4B). These reports suggest the important role of the HDAC3-miRNA network in anti-cancer drug resistance.

## 5. Role of CAGE-miRNA Network in Anti-Cancer Drug Resistance

**Figure 5:**
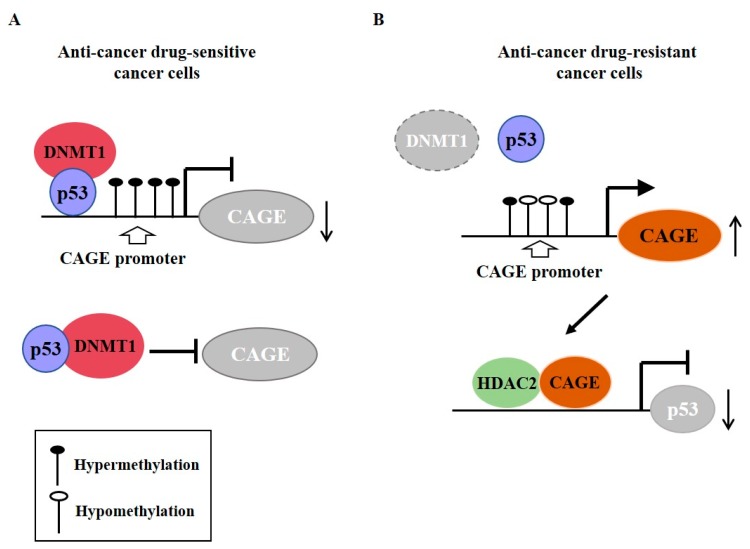
Cancer-associated genes (CAGE) confers resistance to anti-cancer drug resistance. (**A**) In anti-cancer drug-sensitive cancer cells, DNMT1 (DNA methyl transferase I) binds to p53. DNMT causes hypermethylation of promoter sequences of CAGE. In anti-cancer drug-resistant cancer cells, expression of DNMT1 is decreased. This results in hypomethylation of CAGE and increased expression of CAGE. CAGE can bind to HDAC2. The CAGE-HDAC2 complex can bind to the promoter sequences of p53 and decrease expression of p53. The T-bar arrows denote inhibition of transcription/negative regulation and →arrows denote activation of transcription. The ↑arrows denotes increased expression level and ↓arrows denotes decreased expression level.

**Figure 6:**
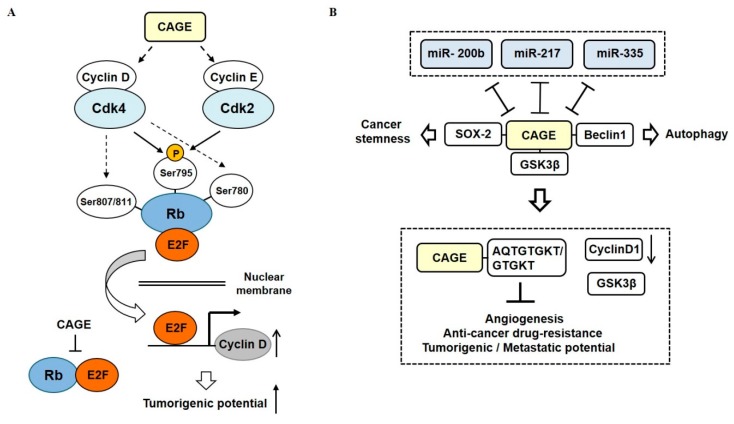
CAGE-miRNA network in anti-cancer drug resistance. (**A**) CAGE increases expression levels of cyclins, which in turn increases cyclin-dependent kinase (CDK) activities. Increased CDK activities phosphorylate Rb to inactivate the Rb protein. Inactivation of Rb leads to the activation of E2 factor (E2F). Activated E2F binds to promoter sequences of cyclins. (**B**) MiR-200b, miR-217, and miR-335 form negative feedback loops with CAGE. CAGE binds to GSK3β, which increases expression of cyclin D1 to confer resistance to anti-cancer drugs. AQTGTGKT, as a CAGE-derived peptide can disrupt the interaction between CAGE and GSK3β and confer sensitivity to anti-cancer drugs. The T-bar arrows denote negative regulation and both side T-bar arrows denote cross inhibition. The hollow arrows denote positive regulation.

Cancer-associated genes (CAGE) have been found in the sera of patients with various cancers [73,74,75]. DNMT1 increases methylation at promoter sequences of CAGE, leading to decreased expression of CAGE [76] (Figure 5A). CAGE can bind to HDAC2 and confers resistance to various anti-cancer drugs [77] (Figure 5B). CAGE can also inactivate tumor suppressor retinoblastoma (Rb) protein by phosphorylation, increase the expression of cyclins, and act as an oncogene [78] (Figure 6A).

CAGE also binds to GSK3β and increases expression of cyclinD, thus conferring resistance to various anti-cancer drugs in melanoma cells [79] (Figure 6B). TargetScan has predicted binding of *miR-200b*, *miR-217*, and *miR-335* to 3′ untranslated regions (UTR) of *CAGE*. *MiR-200b*, *miR-217*, and *miR-335* form a negative feedback loop with CAGE (Figure 6B). CAGE-derived peptides can bind to CAGE and confer sensitivity to anti-cancer drugs in melanoma cells [79] (Figure 6B). CAGE also binds to the sex-determining region y-box 2 (SOX2) and induces cancer stem cell-like properties in melanoma cells [80] (Figure 6B). In addition, CAGE can bind to Beclin1 and induce autophagic flux (manuscript in preparation).

**Figure 7:**
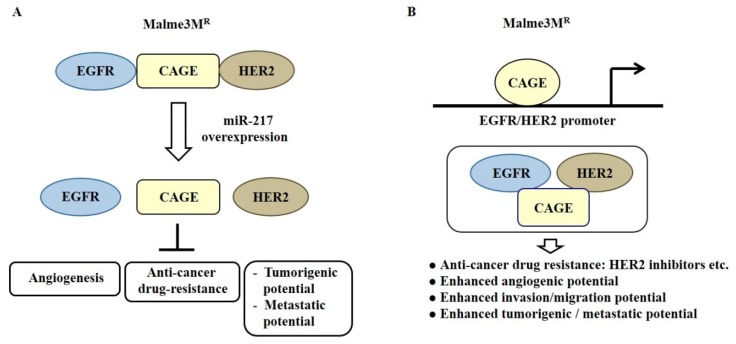
CAGE-miRNA network in anti-cancer drug resistance. (**A**) In anti-cancer drug-resistant cancer cells, CAGE binds to the epidermal growth factor receptor (EGFR) and human epidermal growth factor receptor 2 (HER2). *MiR-217* decreases expression of CAGE and disrupts interactions of CAGE with EGFR and HER2. (**B**) In anti-cancer drug-resistant cancer cells, CAGE binds to the promoter sequences of HER2 and EGFR. This results in increased expression levels of EGFR and HER2. The CAGE-HER2-EGFR complex in Malme3M^R^ cells confers anti-cancer drug resistance. The T-bar arrows denote negative regulation and hollow arrows denote positive regulation.

*MiR-200b* can form a negative feedback loop with CAGE, a cancer/testis antigen, and regulate invasion of a cancer cell line. It also regulates tumorigenic and angiogenic responses to microtubule-targeting drugs [81]. CAGE binds to *miR-200b* and directly regulates expression of *miR-200b* [81]. Recombinant CAGE proteins display angiogenic potential [81]. *MiR-217* can form a negative feedback loop with CAGE and negatively regulates the tumorigenic and metastatic potential of melanoma cells [82]. CAGE binds to the epidermal growth factor receptor (EGFR) and human epidermal growth factor receptor 2 (HER2) and confers in vivo resistance to trastuzumab, an inhibitor of HER2 [82] (Figure 7A). *MiR-217* overexpression prevents interactions of CAGE with EGFR and HER2 in anti-cancer drug-resistant Malme3M^R^ cells [82] (Figure 7A). Down-regulation of EGFR or HER2 can enhance sensitivity to anti-cancer drugs, such as EGFR inhibitors [82]. CAGE directly regulates expression of HER2. It is necessary for the interaction of CAGE with EGFR and HER2 in Malme3M^R^ cells [82] (Figure 7B).

These results indicate that HDAC3 and CAGE can exert an opposite regulation on the expression of *miR-200b* and *miR-217*. This has led us to investigate relationships between HDAC3 and CAGE in anti-cancer drug resistance.

## 6. HDAC3 Functions Upstream of CAGE and Targets CAGE

Anti-cancer drug-resistant cancer cell lines show an increased expression of phosphorylated epidermal growth factor receptor (pEGFR^Y845^) and an interaction between CAGE and EGFR [82]. CAGE-derived GTGKT and AQTGTGKT peptides inhibit activation of EGFR, enhance sensitivity to gefitinib and trastuzumab, and prevent interactions of CAGE with EGFR and HER2 [82]. Vorinostat, an inhibitor of HDACs, decreases expression of EGFR and inhibits renal growth [83]. HDAC3 directly regulates the expression of EGFR by binding to promoter sequences of EGFR, while HDAC inhibitors can reverse the expression of EGFR in colorectal cancer cells [84]. HIF1β activates EGFR and extracellular regulated kinase (ERK) by HDACs [85]. EGFR signaling can increase expression levels of HDACs and inhibit osteoblast differentiation by down-regulating Runt-Related Transcription Factor 2 (Runx2) [86]. These reports imply that HDAC3 might be able to regulate EGFR signaling. 

AG1478, an inhibitor of EGFR tyrosine kinase, inhibits functions of ATP-binding cassette (ABC) transporters, such as ABCB1 and ABCG2 [87]. The targeting of EGFR in cancers is quite limited due to the status of the Kirsten rat sarcoma 2 viral oncogene homolog (KRAS) mutation [88]. KRAS mutants can bypass EGFR to activate Ras/Raf/MEK/ERK signals and significantly weaken the therapeutic effect of cetuximab [89]. C-Met inhibition can enhance sensitivity to anti-cancer drugs, such as 5-Fluorouracil and taxol [90]. Combination therapy employing the inhibition of c-Met and EGFR suppresses tumorigenic potential of hepatocellular carcinoma cells [91]. These reports suggest that HDAC3 may regulate the expression of CAGE and the activation of EGFR in anti-cancer drug-resistance.

**Figure 8:**
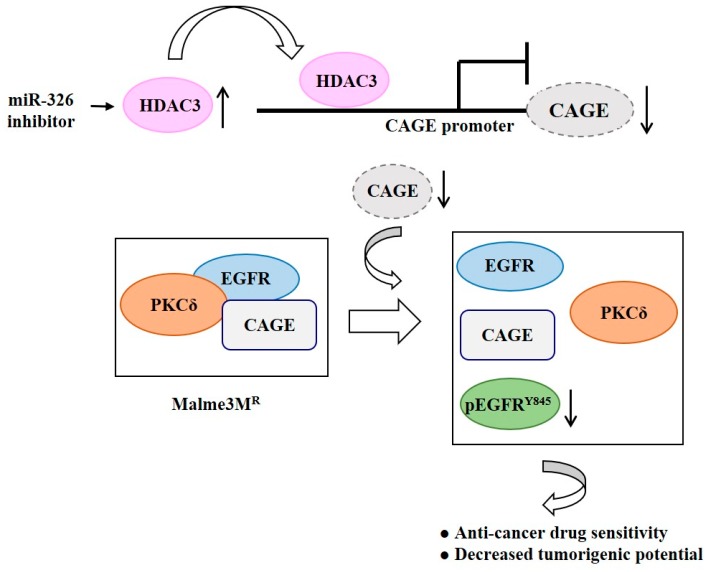
HDAC3 targets CAGE to inhibit EGFR activation. The *MiR-326* inhibitor increases expression of HDAC3, which in turn induces binding of HDAC3 to the promoter sequences of CAGE. Decreased expression of CAGE leads to a disruption of interactions of CAGE with EGFR and protein kinase C gamma (PKCδ) and decreased expression of pEGFR ^Y845^ in Malme3M^R^ cells. The ↑arrows denotes increased expression level and ↓arrows denotes decreased expression level.

The *MiR-326* inhibitor increases the expression of HDAC3, which results in the binding of HDAC3 to promoter sequences of CAGE in anti-cancer drug-resistant Malme3M^R^ cells (Figure 8). Decreased expression of CAGE leads to enhanced sensitivity to anti-cancer drugs [69] (Figure 8). HDAC3 negatively regulates the expression of pEGFR^Y845^ (Figure 8). Therefore, HDAC3 can target CAGE to regulate the activation of EGFR signaling, responses to various anti-cancer drugs, and the tumorigenic potential of cancer cells. 

## 7. Discussion and Conclusions

Reports from our laboratory and others indicate that HDAC3 regulates responses to anti-cancer drugs, tumorigenic potential, and angiogenesis. MicroRNAs that regulate the expression of HDAC3 can also regulate responses to anti-cancer drugs, and the tumorigenic potential and angiogenic potential of cancer cells. Our studies provide the mechanism of anti-cancer drug resistance regulated by HDAC3. We discovered the role of CAGE, a cancer/testis antigen, in anti-cancer drug resistance and the associated mechanisms. 

MicroRNAs that form negative feedback loops with CAGE can regulate responses to anti-cancer drugs and form positive feedback loops with HDAC3. HDAC3 functions upstream of CAGE and negatively regulates expression of CAGE. CAGE-promoted anti-cancer drug resistance involves direct regulation of EGFR and HER2 by CAGE and interactions of CAGE with EGFR and HER2. 

To overcome anti-cancer drug-resistance, it might be necessary to employ miRNA-inhibitors or miRNA-mimics that can increase expression of HDAC3 while decreasing expression of CAGE. It would be also necessary to identify small molecules that could increase expression of HDAC3. For this, it would be necessary to develop screening tools based on the structure of HDAC3. For better understanding of anti-cancer drug resistance regulated by HDAC3, it would be necessary to employ cancer patient-derived tissues and cell lines that show low level expression levels of HDAC3 but high expression of CAGE. This approach will give better understanding of anti-cancer drug resistance and strategy to develop anti-cancer drugs. Overall, HDAC3 can serve as a target for developing anti-cancer drugs and/or drugs that can enhance sensitivity to anti-cancer drugs in clinical use.
